# Clinical experience with navigated 3D ultrasound angiography (power Doppler) in microsurgical treatment of brain arteriovenous malformations

**DOI:** 10.1007/s00701-016-2750-3

**Published:** 2016-03-19

**Authors:** Geirmund Unsgård, Vidar Rao, Ole Solheim, Frank Lindseth

**Affiliations:** Neurosurgical Department, St. Olav University Hospital, Trondheim, Norway; Norwegian University of Science and Technology, Trondheim, Norway; SINTEF, Trondheim, Norway

**Keywords:** AVM, Microsurgery, Clipping of feeders, Navigated 3D US angiography

## Abstract

**Introduction:**

We have previously described a method that has the potential to improve surgery of arteriovenous malformations (AVMs). In the present paper, we present our clinical results.

**Materials and methods:**

Of 78 patients referred for AVMs to our University Hospital from our geographical catchment region from 2005 through 2013, 31 patients were operated on with microsurgical technique. 3D MR angiography (MRA) with neuronavigation was used for planning. Navigated 3D ultrasound angiography (USA) was used to identify and clip feeders in the initial phase of the operation. None of our patients was embolized preoperatively as part of the surgical procedure. The niduses were extirpated based on the 3D USA. After extirpation, controls were done with 3D USA to verify that the AVMs were completely removed. The Spetzler three-tier classification of the patients was: A: 21, B: 6, C: 4.

**Results:**

Sixty-eight feeders were identified on preoperative MRA and DSA and 67 feeders were identified and clipped by guidance of intraoperative 3D USA. Six feeders identified preoperatively were missed by 3D USA, while five preoperatively unknown feeders were found and clipped. The overall average bleeding was 440 ml. There was a significant reduction in average bleeding in the last 15 operations compared to the first 16 (340 vs. 559 ml,* p* = 0.019). We had no serious morbidity (GOS 3 or less). New deficits due to surgery were two patients with quadrantanopia (one class B and one class C), the latter (C) also acquired epilepsy. One patient (class A) acquired a hardly noticeable paresis in two fingers. One hundred percent angiographic cure was achieved in all patients, as evaluated by postoperative DSA.

**Conclusions:**

Navigated intraoperative 3D USA is a useful tool to identify and clip AVM feeders. Microsurgical extirpation assisted by navigated 3D USA is an effective and safe method for removing AVMs.

## Introduction

The reported results from the various interventions for brain AVMs are heterogeneous [13]. In spite of the large number of publications about embolization and Gamma Knife treatment of AVMs in recent years, the most efficient treatment in terms of radiological cure still is microsurgical extirpation. According to a meta-analysis from 2000 to 2011, successful brain AVM obliteration was achieved in 96 % after microsurgery, 38 % after stereotactic radiosurgery, and 13 % after embolization [13]. Gamma Knife therapy is often preferable in deep-seated lesions with difficult locations because the AVM can be occluded with less risk of complications than with microsurgical treatment [3]. Embolization has, because of the low occlusion rate, mostly been used in combination with surgery to reduce the challenge for the surgeon. Many AVMs, especially those with Spetzler three-tier class A (Spetzler/Martin grade I and II), are treated by microsurgery only [10]. AVM surgery is challenging. We have used ultrasound technology to assist in the resection of these difficult lesions with the intention to reduce the harm to the patients. In 2005, we published a paper describing the feasibility of using a navigation system with stereoscopic display of the vessels and 3D ultrasound angiography (USA) to identify and occlude feeding vessels in the initial phase of the operation [12]. In the present paper, we summarize our clinical experience with this technology.

## Materials and methods

### Patients

Norway has a socialized and regionalized health care system where all patients with neurosurgical diseases in a defined geographical catchment region are referred to the serving university hospital. In the period from 2005 through 2013, 78 patients with AVMs were referred to our neurosurgical department. Before deciding the treatment, patients and their closest family were thoroughly informed about the different treatment modalities. Thirty-three patients with small AVMs in deep-seated locations were referred to Gamma Knife treatment. Thirty-one were treated with a microsurgical operation. Fourteen were not offered treatment because of high risk (S/M grade V), high age, and significant co-morbidity or due to patient preferences.

The incidence of microsurgically operated AVMs in our region, with 700,000 inhabitants, is accordingly around 0.5 per 100,000 per year. The annual incidence rate of diagnosed AVMs is 1.2 per 100,000.

All patients underwent both DSA and MRA as part of the diagnostic work up. Some of the patients with AVMs in eloquent areas also underwent fMRI and/or MR tractography. In all operations, we used navigated 3D USA, as described earlier [12]. The Regional Ethical Committee approved the study, and the patients signed an informed consent form. The operated patients were all untreated except for two. One had at a previous occasion been treated with embolization three times together with Gamma Knife treatment. Another had been treated with Gamma Knife. Both were referred to surgery due to treatment failure. None of the 31 operated patients was embolized preoperatively as part of the surgical procedure. Demographics and the results for the operated patients are shown in Tables 1 and 2. The patients were, before surgery, graded according to the Spetzler/Martin system. In this paper we will use the Spetzler three-tier classification, where S/M grade I and II is combined to a class A, S/M grade III equals class B, and S/M grade IV and V is combined to a class C [10]. The classifications of the patients were: 21 class A (8 S/M grade I and 13 S/M grade II), six class B, and four class C (S/M grade IV). The causes for diagnostic investigations were headache/dizziness/fatigue – 12 cases, ICH – eight cases, epilepsy – six cases, neurological deficits – two cases, no symptoms related to the AVM – three cases. In 16 of the 31 cases the AVM were located in eloquent parts of the brain.

**Table 1:** Grading, eloquence, and intraoperative events

Spetzler three-tier class (10)	Patients (*n*)	Sex (male/female)	Age (years)	Feeders visualized preoperative (#)	Feeders clipped before resection (#)	Duration of operation (min)	Perop. bleeding (ml)	Eloquent* n* (%)
**A (I/II)**	21	9/12	41.5	43	40	303	379	7 (33)
**B (III)**	6	4/2	46.3	15	20	355	391	5 (83)
**C (IV)**	4	2/2	56.8	10	7	443	837	4 (100)
**Total**	**31**	**15/16**	**44.4**	**68**	**67**	**331**	**440**	**16 (52)**

**Table 2:** Preoperative symptoms and postoperative outcome

Spetzler three-tier class (10)	Patients (*n*)	Preop. symptoms* n* (symptoms)	Postop. complications* n* (complications)	Status 3 days postop.* n* (symptoms)	Status 3 months postop.* n* (patients)
**A I/II**	21	Asymptomatic 3 Headaches/Dizziness 8 Hemianopia 3 ICH 6 Epileptic seizure 4 Hemiparesis 2 Paresthesia 1	None 15 PE 1 Hematoma 2 Pneumonia 3 Epileptic seizure 2 UTI 1	Normal 14 Paresis 3 Aphasia 3 Ataxia 1 Hemianopia 2	Normal 18 Unchanged deficit 2 New deficit 1 (feeling of less control in two fingers)
**B III**	6	Headaches/Dizziness/Fatigue 4 ICH 1 Epileptic seizure 1 Hemiparesis 1	None 5 UTI 1	Normal 3 Aphasia 2 Hemianopia 1	Normal 4 Unchanged deficit 1 New deficit 1 (lower quadrantanopia)
**C IV**	4	Headaches/Dizziness/Fatigue 3 ICH 1 Epileptic seizure 1	None 2 Epileptic seizure 2	Paresis 1 Ataxia 1 Hemianopia 2	Normal 2 Unchanged deficit 1 New deficit 1 (lower quadrantanopia and epilepsy)
**Total**	**31**				

*ICH* intracerebral hematoma,* PE* pulmonary embolism,* UTI* urinary tract infection

New deficit includes both neurological deficit and epilepsy

### Navigation equipment

**Fig. 1:**
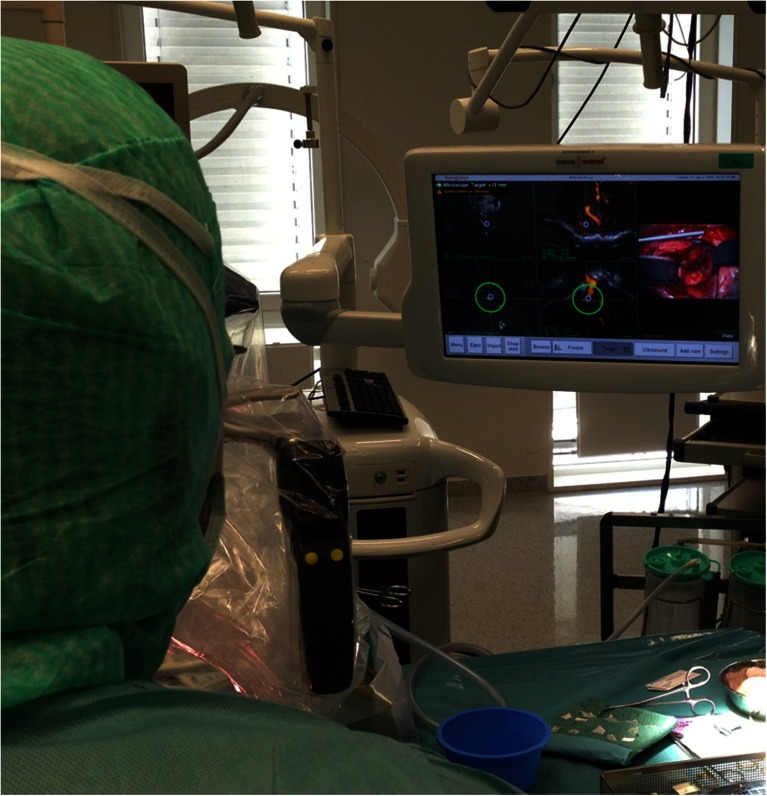
The setup in the operation room. The navigation/intra-operative imaging system (SonoWand Invite) is placed to the right of the surgeon. The monitor shows that the target point is set on the feeder. The* upper planes* on the monitor are planes along the visual line of the microscope and the* lower planes* are perpendicular to the visual line through the target point

The preoperative 3D MRA was imported into the ultrasound-based intraoperative imaging system SonoWand (Sonowand A/S, Trondheim, Norway). Power Doppler-based 3D USA was obtained during the procedure. Until 2009 we used version 1.4 of the SonoWand system as described in a previous paper [12]. After that we have used the SonoWand Invite system. In the Invite version, there is no stereoscopic module available, so in addition we used the research system Custus X, which provided rendering of both 3D MRA and 3D USA. Since 2012 we have used navigated microscope and a targeting function (Fig. 1). The target point is set by a navigator (rotation sensitive pointer) with offset. With the navigated microscope, a plane with the visual line (center of the microscope vision down to focus point) and a plane perpendicular to this plane through the target point appear as navigated cross sections on the navigation monitor (tip view). A dotted line extending the visual line indicates the distance from the focus point of the microscope to the target point.

### The procedure

**Fig. 2:**
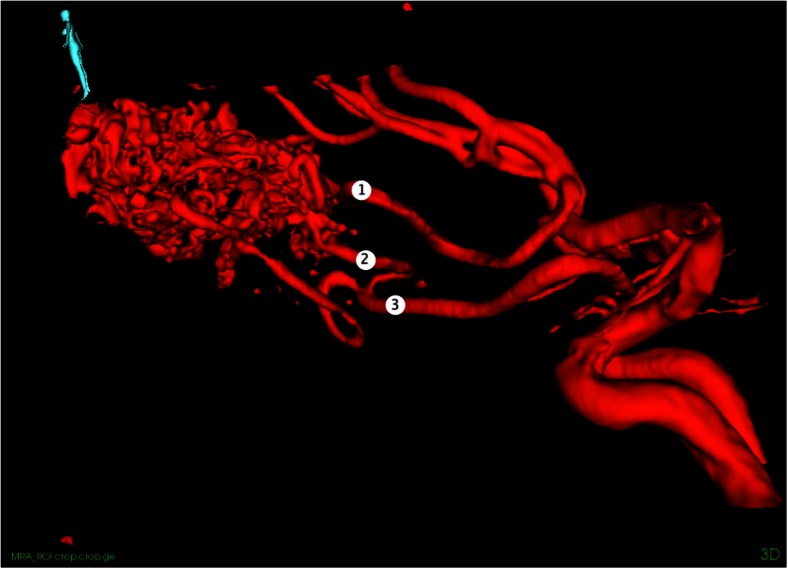
Rendering of MRA of a fronto basal AVM showing three locations where we clipped feeders. Pericallosal vessels are seen in the upper part of the figure

**Fig. 3:**
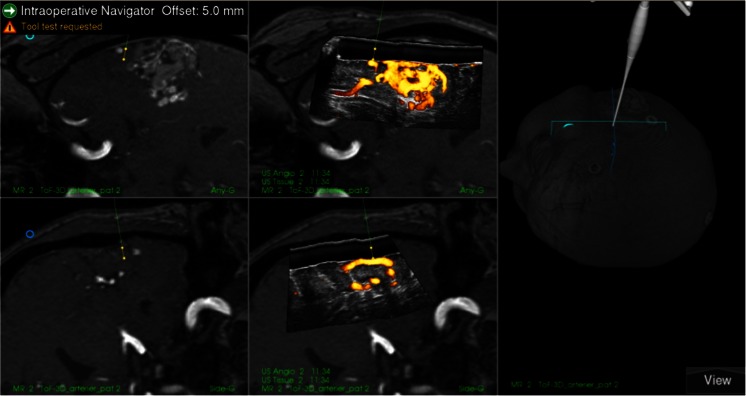
Snapshot of the navigation monitor. Preoperative 3D MRA registered to the patient’s head is in the* right column* and an intraoperative 3D USA volume inserted in the 3D MRA volume is in the* middle column*. Cross sections of the volumes are displayed in dual anyplane steered by a rotation sensitive navigator. The images in the* upper row* are close to axial view, and the images in the* lower row* are perpendicular to the upper images through the virtual extension line of the navigator. The navigator with 5-mm offset is pointing at a feeder in the 3D USA volume. The feeder is missed in the 3D MRA volume

**Fig. 4:**
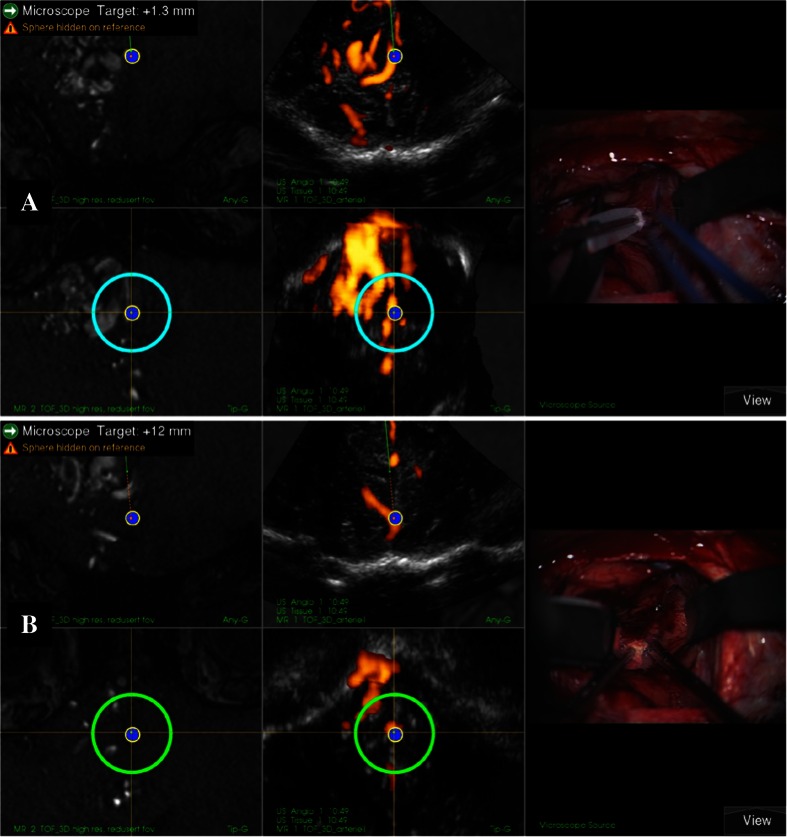
Two different snapshots of the navigation monitor (**a** and **b**). The* middle column* in **a** shows the target point for clipping of feeder 1 of the front basal AVM shown in Fig. 2. The* middle column* in **b** shows the target point for clipping of feeder 2 in Fig. 2.* Left columns* show the corresponding MRAs. The visual line of the microscope is set on the target point in both **a** and **b**. The* dotted line* and the* text in the upper left corner *indicate the distance from the microscope focus to the target point

As the first step during surgeries, the 3D MRA were imported into the navigation system and registered to the head of the patient. Based on the 3D MRA, the head was adjusted to facilitate a horizontal position of the craniotomy, which is associated with less brain shift during surgery and is better for obtaining good ultrasound 3D image recordings [11]. The stereoscopic display or the 3D MRA rendering was used for updating the surgeon on the angiographic architecture of the AVM. It was also used for detailed planning of where to occlude the different feeders (Fig. 2). After the craniotomy, a 3D USA volume was recorded by moving the appropriate probe with optical tracking over the area of interest. The image volumes were usually displayed in dual anyplane, the upper plane being a cross section steered either by a rotation-sensitive navigator or a microscope, and the lower plane being a cross section perpendicular to the upper plane along the extension line of the navigator or the centerline of the microscope view. A shift between 3D MRA and 3D USA was observed in most patients (Fig. 3). 3D MRA was used for facilitating interpretation of the 3D USA, but due to the shift/registration inaccuracy of the 3D MRA, the identification and clipping of the feeders were done based on the 3D USA data. As a tool for identification of the feeders, we either used a navigator or the navigated microscope. In cases with navigated microscope, the target point was set with a navigator (with offset) at the planned point for clipping of the feeder (Fig. 4). The upper row is the same cross section as in dual anyplane. After having set the target point, the lower row shows a plane perpendicular to the visual line of the microscope (“tip view”). After clipping of the feeders, a new 3D USA was acquired to look for and eventually clip additional feeders. After resection of the nidus, a final 3D USA was acquired as a resection control.

## Results

### Identification and clipping of feeders

In the 31 patients, a total of 68 feeders were identified from preoperative DSA and 3D MRA. From 3D USA recordings, 67 feeders were also identified and subsequently clipped but not always the same feeders that had been identified on the preoperative images. A concordance between 3D MRA/DSA and 3D USA was found for 62 feeders (91 %). Six feeders identified in the preoperative MRA/DSA were missed by 3D USA. In one patient, as many as four very thin feeders were visualized in preoperative DSA/MRA that we were unable to identify in the 3D USA images. That patient had, at a previous occasion, been treated with embolization three times together with Gamma Knife treatment. On the other hand, 3D USA identified five feeders that were not seen in MRA/DSA, four of them in the 2nd 3D USA recorded after the initial clipping.

### Resection of the AVMs

**Fig. 5:**
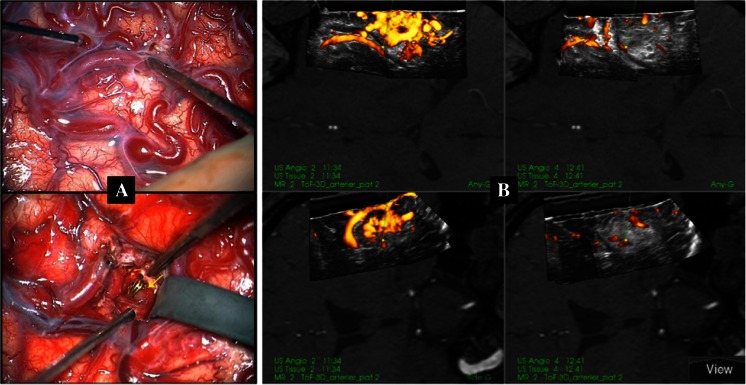
**a** The microscope images before (*upper image*) and after clipping of a deep-seated feeder (*lower image*,* yellow arrow*). **b** Snapshot from the navigation monitor with 3D USA displayed in dual anyplane (described in legend to Fig. 3).* Column to the left* is before clipping.* Column to the right* is after having placed the AVM clip on the feeding vessel as shown in the microscope image (*yellow arrow*)

**Fig. 6:**
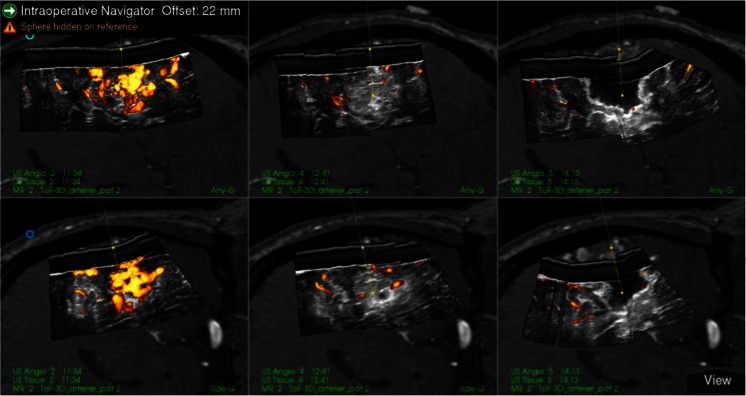
Snapshot from the navigation monitor showing 3D USA in dual anyplane. This AVM had three feeding vessels.* Column to the left* shows the flow before clipping any of the feeders. The* column in the middle* shows the flow after clipping the three feeders. The* column to the right* is a resection control 3D USA showing that the nidus is completely removed. The resection was assisted by navigation based on the 3D USA volume acquired before clipping the feeders (*column to the left*)

After clipping of all the identified feeders, a new 3D USA recording showed in some cases large reduction of power Doppler signals in the nidus indicating low flow (Figs. 5 and 6). In those cases, it was very easy to resect the AVMs with very little blood loss. In many cases, there was still considerable flow after clipping, though much reduced as evaluated by the turgor of the AVM nidus and the 3D power Doppler signal in the nidus. This remnant flow was presumably due to small feeding vessels (perforators) that were not identified and clipped in the early phase of the operation.

3D USA was also helpful in defining the borders of the AVM during extirpation. In cases where the power Doppler signal after clipping was reduced to nearly nothing in the nidus, the 3D USA acquired before the clipping of the feeders was used for accurate resection of the nidus (Fig. 6).

The average peroperative bleeding for all cases was 440 ml. For the 10 class B and C cases, the average bleeding was 579 ml. For the first 16 patients, including 2 class B and 3 class C cases, the average bleeding was 559 ml, and in the last 15 cases including 4 class B and one class C, the average bleeding was 340 ml (*p* = 0.019 by the Mann–Whitney* U* test). This may indicate some improvement during the development of the technology.

At the end of the resection, a new 3D USA acquisition confirmed that the AVM was completely removed in all the cases (Fig. 6).

### Outcome

The outcomes are summarized in Table 2. Postoperatively, four patients had early and transient epileptic seizures that were handled without the need for long-term antiepileptic medication. One patients had pulmonary embolisms, two had a postoperative surgical site hemorrhage, and three had postoperative pulmonary or urinary tract infections. None of the patients were reoperated and all these postoperative adverse events were handled without permanent loss of functions or health.

As for permanent neurological deficits (after 3 months) two patients had acquired a homonym quadrantanopia as a consequence of the operation. One was a class B and the other was a class C patient. The latter also acquired epilepsy 2 months after the operation. One patient with a class A AVM in the precentral gyrus by the hand knob had no evident paresis after 3 months, but still he had a feeling of reduced fine motor control in two fingers. One patient with a class A AVM that had been treated with Gamma Knife radiosurgery 4 years earlier had no deficit 3 months after the operation, but 10 months after the operation she had an epileptic seizure. One patient with a large ICH at presentation had a hemiparesis that was temporarily worsened after the operation. At control 3 months later he was back to his preoperative status.

All patients underwent DSAs 3 months after the operation. No residual AVM and normalization of the vasculature were confirmed in all cases.

## Discussion

The technique of operating AVMs with early clipping of feeders based on neuronavigation was reported many years ago [8]. However, the identification of the feeders based on navigation in preoperative MR angiograms can be difficult because of the registration error and the brain shift. We have in a previous article demonstrated the concept of using navigated 3D US angiography to identify both superficial and deep feeders [12]. The present consecutive case series is a follow-up of our clinical results.

In a medium-sized neurosurgical unit, AVM operations are uncommon, with limited possibilities for neurosurgeons to obtain experience with these challenging patients. Therefore, every development that can make this operation easier should be acknowledged.

One technique that has been used for some time in AVM surgery is indocyanine green angiography. We have no experience with this modality but according to a recent paper from one of the most merited groups in AVM surgery, it does not improve the clinical outcome and it seems to have shortcomings in deep AVMs [14].

Our concept is somewhat different to the usual way of operating AVMs and can be summed up as follows: Navigated 3D MRA is registered to the patient’s head and rendered for planning of the craniotomy and where to clip the different feeding vessels. DSA is used in the preoperative work-up to confirm the 3D MRA findings of feeders and draining veins. The 3D USA can be recorded both before and after opening of the dura. The identification of the feeding vessels should be done based on the latter image volume, because there is often a small shift between navigated 3D USA acquired before and after opening of the dura. Before even touching the nidus, feeding vessels are identified, localized, and clipped. The identification of feeders based on 3D USA is reliable. Only six of 68 feeders identified on preoperative DSA/MRA were missed. Four of these feeders were in one patient that earlier had been treated three times with embolization and also have had Gamma Knife treatment. The feeders in this patient were tiny and hard to recognize also in the MRA volume. The strength of the method is that even very deep-seated feeders can be reached and identified with precise transcortical dissection by navigated 3D USA before starting the dissection of the nidus. Navigation towards the feeders is facilitated by navigated microscope. By moving the microscope to a position where the navigated centerline of the microscope hit the target point of the feeder, a precise dissection through the brain and identification of the feeder can be done by using the crosshairs of the microscope.

When all the feeders have been clipped, a second 3D USA volume is acquired to recognize residual flow in the nidus and possibly unidentified feeders. This second 3D USA after clipping and before starting the resection was done routinely only in the last 15 cases. In four cases, additional feeding vessels not identified in the preoperative work-up were discovered and clipped. Presumably, the vessels were discovered because the major part of the flow was eliminated from the nidus.

In most cases, the turgor and the power Doppler activity in the nidus was strongly reduced after clipping, but in a few cases, there was still cumbersome bleeding due to small feeders that could not be properly identified neither from the preoperative MR angiographies, DSAs, nor in the intraoperative 3D US angiograms. A navigated resection was done guided by the 3D USA volume of the nidus acquired before start of the clipping of feeders [6].

Resection control with 3D USA after removal of the nidus was done in all cases to confirm complete removal of the AVM. No intraoperative or postoperative DSA was done until the DSA control 3 months after the surgery.

One limitation of the technology is the lack of information about the direction of blood flow in the power Doppler angiography. A method has been developed for mapping angle-corrected velocities and flow direction to the 3D geometry of the vessels [4, 5], but it has not been available in the operation theatre for the cases presented in this study. Thus, the identification of feeders vs. veins has been based on the angioarchitecture and the comparing of intraoperative 3D USA to the preoperative 3D MRA. This way of identifying feeders vs. veins was reliable in this material, because we never misinterpreted the nature of the vessels. But it claims some concern from the surgeon. Our method for mapping flow direction to the 3D geometry of the 3D USA is now available in the operation theatre, and will be used for future AVM operations.

The planning and clipping procedure added some extra time to the fist phase of the operation. The average operation time is 320 min for all operations and 274 min for the last 15 operations, which by some will be considered long. On the other hand, operation time was not an issue in this series, which also involved some other research tasks not reviewed in this paper.

We did not use preoperative embolization for any of our patients. One of the patients had been unsuccessfully embolized years earlier at another hospital. Our assumption is that what can be gained with preoperative embolization in terms of making the extirpation of the nidus easier can be achieved by navigated intraoperative clipping of the feeding vessels without the extra risks associated with embolization. Moreover, incompletely embolized vessels may be a problem during subsequent surgery [3]. There may be cases where large, deep-seated feeding vessels are better handled with preoperative embolization, but in our cohort there were no such cases. There would certainly be a possibility to combine the two techniques since it is unlikely that the embolization of a deep feeder would interfere much with the image quality of the power Doppler angiography.

In only 25 % of our patients the AVMs were diagnosed because of hemorrhage. The way of handling unruptured AVMs is controversial. Studies have indicated that patients with unruptured AVMs can be handled with conservative, medical management instead of interventional therapy [1, 7]. These results have been criticized[2], and a recent paper from a group with a large experience in AVM surgery concludes that surgery should be regarded as the first-line or gold-standard therapy for the majority of low-grade (class A) AVMs [9]. An unknown factor is the quality of life for a patient harboring an AVM with a bleeding potential. Thus, surgical resection seems to be an optimal handling also of patients with unruptured AVMs, as long as we are able to keep the complication rate at a low level.

In a recent paper, serious procedural complications in AVM surgery were defined as GOS equal or less than 3 after 30 days [3]. Using that definition, none of our patients experienced serious procedural complications.

Considering neurological deficits, the only acquired deficit in the class A or low-grade AVM patients was one patient with an AVM in the precentral gyrus that had a feeling of reduced fine motor control of two fingers evaluated 3 months after the operation. It had no influence on his daily activity and can hardly be registered as morbidity. In a recent publication, a summation of the results of 12 published series showed a morbidity for low-grade AVMs of 2.2 % [9]. The numbers of class B (6) and C (4) patients are too small for a reasonable evaluation. One class B patient acquired quadrantanopia and one class C patient acquired both a quadrantanopia and epilepsy. The quadrantanopia seemed to be of little concern to these patients, but unfortunately we do not have quality-of-life data.

The effectiveness of the treatment is excellent since postoperative DSAs confirmed no residual AVM in any of the patients. The 100 % radiological cure is probably facilitated by active use of 3D US angiography during the operations.

## Conclusions

Navigated intraoperative 3D USA is a helpful tool to identify, localize, and clip AVM feeders. Good clinical and excellent radiological results can be obtained by microsurgical extirpation assisted by navigated intraoperative 3D US angiography.
